# Role of Gender in the Prevalence of Myopia among Polish Schoolchildren

**DOI:** 10.1155/2019/9748576

**Published:** 2019-07-02

**Authors:** Maciej Czepita, Damian Czepita, Krzysztof Safranow

**Affiliations:** ^1^2nd Department of Ophthalmology, Pomeranian Medical University, al. Powstańców Wlkp. 72, 70-111 Szczecin, Poland; ^2^Department of Biochemistry and Medical Chemistry, Pomeranian Medical University, al. Powstańców Wlkp. 72, 70-111 Szczecin, Poland

## Abstract

**Purpose:**

The aim of the paper was to study the role of gender in the progression of myopia among Polish schoolchildren.

**Materials and Methods:**

4875 children from elementary schools and high schools were examined (2470 boys, aged 6–16 years, mean age 11.0, SD = 2.6 and 2405 girls, aged 6–16 years, mean age 11.1, SD = 2.6). The examined students were Caucasian and resided in and around Szczecin, Poland. The examination included retinoscopy under cycloplegia. The refractive error readings were reported as spherical equivalent (SE). Myopia was defined as SE of at least −0.5 D. Data analysis was performed using the Mann–Whitney *U* test and 2-sided Fisher's exact test. *p* values of less than 0.05 were considered statistically significant.

**Results:**

It was found that the SE among Polish boys is similar to the SE among Polish girls before the age of 9 years. However, in older children, lower SE values and higher prevalence of myopia were found among girls than boys, both at 9–13 years range (0.45 ± 1.05 vs 0.55 ± 1.23 D, *p*=0.047 and 8.30% vs 5.71%, *p*=0.015, respectively) and at 13–16 years range (0.32 ± 1.14 vs 0.54 ± 1.08 D,  *p*=0.0093 and 10.37% vs 5.96%, *p*=0.0050), respectively.

**Conclusions:**

Gender is associated with the prevalence of myopia among Polish schoolchildren ranging from 9 to 16 years of age.

## 1. Introduction

Several studies have been carried out in different countries on the role of gender in the progression of myopia among schoolchildren. In Poland, only one paper dealing with the issue has been published [1–12].

Several contradictory results from these studies can be found in the world literature. However, most researchers point to a more frequent occurrence of myopia in girls [1–6, 9, 11, 12] than in boys [7, 8] (Table 1).

Due to the discrepancies in the obtained data, we decided to examine the spherical equivalent (SE) on a large population of 4875 Polish students after cycloplegia with 1% tropicamide.

## 2. Materials and Methods

The studies were carried out from October 2000 to March 2009. 4875 children from elementary schools and high schools were examined (2470 boys, aged 6–16 years, mean age 11.0, SD = 2.6 and 2405 girls aged 6–16 years, mean age 11.1, SD = 2.6). The examined students were Caucasian and resided in and around Szczecin, Poland.

Twenty-one schools were selected by random sampling out of 210 schools from the area of Szczecin. All children from the selected schools were invited to participate in the study. However, only 95.8% accepted to participate. We did not observe differential dropout.

Every examined student had undergone the following examinations: distance visual acuity testing, cover test, anterior segment evaluation, and cycloplegic retinoscopy after instillation of 1% tropicamide, and a questionnaire was taken. The methodology of the examinations has been described in detail in previous work.

Data analysis was performed using the Mann–Whitney *U* test and 2-sided Fisher's exact test. *p* values of less than 0.05 were considered statistically significant [13].

## 3. Results

**Figure 1:**
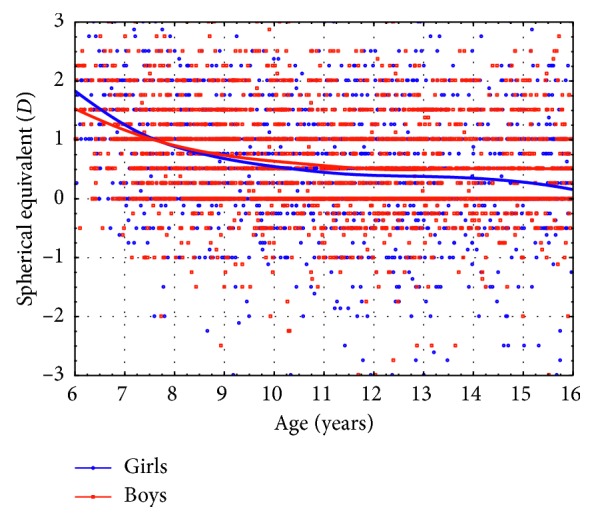
Spherical equivalent in relation to the age of boys (red line) and girls (blue line). Regression lines are obtained using distance-weighted least squares fitting method.

It was found that the spherical equivalent among Polish boys is similar to the SE among Polish girls before the age of 9 years. However, in older children, lower SE values and higher prevalence of myopia were found among girls than boys, both at 9–13 years range (0.45 ± 1.05 vs 0.55 ± 1.23 D, *p*=0.047 and 8.30% vs 5.71%, *p*=0.015, respectively) and at 13–16 years range (0.32 ± 1.14 vs 0.54 ± 1.08 D, *p*=0.0093 and 10.37% vs 5.96%, *p*=0.0050), respectively (Figure 1, Tables 2 and 3).

## 4. Discussion

It is widely known that myopia occurs more often in pupils who spend a lot of time reading, writing, or using a computer [13–15]. Myopia occurs less often in pupils who spend a lot of time doing outdoor activities [13, 14, 16]. It is widely regarded that myopia occurs more often in girls than in boys, especially in older children. In our study, we also observed a higher occurrence of myopia in girls aged 9 to 16 years. A similar relationship was observed by other authors. Only Maul et al. [8] in Chile concluded that myopia occurs more often in boys aged 5–15 years.

In order to reduce the possibility of making a mistake, we decided to conduct the examinations on a large population of 4875 students after cycloplegia with 1% tropicamide. Besides, the examinations were performed only by two doctors. According to Zadnik et al. [17], 95% limits of agreement for cycloplegic retinoscopy are ±0.95 D.

Based on the conducted examinations, we found that in Polish schoolchildren, with age, a decrease in the spherical equivalent occurs. A faster and larger decrease was observed in girls compared to boys. This may indicate that myopia occurs earlier and more often in girls than in boys. In 2007, we published a similar paper on the prevalence of refractive errors among children aged 6–18 years. We concluded that the prevalence of myopia among boys was 5.1% and among girls was 7.4% [2].

The data obtained by us are similar to the results of investigations performed in India [1], Poland [2], Armenia [3], Malaysia [4], Taiwan [5], Australia [6], Finland [9], Nepal [10], Singapore [11], and China [12]. However, they differ from the results gathered in Hong Kong [7] and Chile [8].

It is widely accepted that there are two possibilities for gender differences. The first is that the differences are biologically determined. The second possibility is that they are socially/behaviorally determined.

Zylbermann et al. [18] determined that Orthodox Jewish boys, who receive an intensive religious education, are much more myopic than their sisters and the rest of their age cohort who receive a more secular education. Probably, the high degree and prevalence of myopia observed in the Orthodox male group may be due to their heavy accommodative eye use attributed to their different study habits.

Recent extensive studies carried out in China on the prevalence of myopia have concluded that myopia occurs more often in girls. Ma et al. [19] have shown that myopia occurs more often in girls below 3 years of age. However, Li et al. [20] concluded that myopia occurs more often in 12.7-year-old girls.

According to Krause et al. [21], the reasons for sex differences are determined by genetic factors, dietary factors, and amount of close work, as well as are connected with puberty. Girls reach puberty earlier than boys and therefore reach their final body height one or two years earlier than boys. This leads to a rise in the prevalence of myopia.

Our results are similar to the results obtained by other authors. We also demonstrated that gender is associated with the prevalence of myopia.

## 5. Conclusions

Gender is associated with the prevalence of myopia among Polish schoolchildren ranging from 9 to 16 years of age.

## Figures and Tables

**Table 1:** Dependency between gender and myopia.

Reference	Country	Time of data collection (years)	Age (years)	Prevalence of myopia		Girls and boys (%)
				Girls (%)	Boys (%)	
Ahmed et al. [1]	India	2007	6–22	5.4	3.6	1.8
Czepita et al. [2]	Poland	2000–2005	6–18	7.4	5.1	2.3
Giloyan et al. [3]	Armenia	2011	10–16	53.4	46.6	6.8
Goh et al. [4]	Malaysia	2003	7–15	21.2	17.5	3.7
Hsu et al. [5]	Taiwan	2005-2006	7–13	25.9	25.3	0.6
Ip et al. [6]	Australia	2003–2005	11–15	14.1	9.7	4.4
Lam and Goh [7]	Hong Kong	1990-1991	6–17	55.9	57.4	−1.5
Maul et al. [8]	Chile	1998	5–15	14.7	19.4	−4.7
Mäntyjärvi [9]	Finland	1980-1981	7–15	26.6	19.5	7.1
Pokharel et al. [10]	Nepal	1980-1981	5–15	1.5	1.5	0
Quek et al. [11]	Singapore	2002	15–19	72.7	67.7	5.0
Zhao et al. [12]	China	1998	5–15	23.5	14.1	9.4

**Table 2:** Spherical equivalent (*D*) among examined boys and girls.

Age (years)	Boys	Girls	*p* ^*∗*^
	Mean ± SD	Mean ± SD	
6–9 (≥6 and <9)	+0.95 ± 1.04	+0.99 ± 1.21	0.91
9–13 (≥9 and <13)	+0.55 ± 1.23	+0.45 ± 1.05	0.047
13–16 (≥13 and <16)	+0.54 ± 1.08	+0.32 ± 1.14	0.0093

SD: standard deviation. ^*∗*^Mann–Whitney *U* test.

**Table 3:** Prevalence of myopia defined as spherical equivalent of at least −0.5 D among examined boys and girls.

Age (years)	Boys (%)	Girls (%)	*p* ^*∗*^
	(95% CI)	(95% CI)	
6–9 (≥6 and <9)	3.65 (2.35–5.38%)	3.35 (2.11–5.03%)	0.88
9–13 (≥9 and <13)	5.71 (4.46–7.18%)	8.30 (6.76–10.07%)	0.015
13–16 (≥13 and <16)	5.96 (4.23–8.12%)	10.37 (8.08–13.05%)	0.0050

95% CI: 95% confidence interval. ^*∗*^Fisher's exact test.

## Data Availability

The data used to support the findings of this study are available from the corresponding author upon request.
